# Machine Ethics: Do Androids Dream of Being Good People?

**DOI:** 10.1007/s11948-023-00433-5

**Published:** 2023-03-23

**Authors:** Gonzalo Génova, Valentín Moreno, M. Rosario González

**Affiliations:** 1 Computer Science and Engineering Department, Universidad Carlos III de Madrid, Avda. Universidad 30, 28911 Leganés, Madrid, Spain; 2 Department of Educational Studies, Universidad Complutense de Madrid, Avda. Rector Royo Vilanova S/N, 28040 Madrid, Spain

**Keywords:** Rationality of ethics, Machine ethics, Moral codes of conduct, Intentional action, Artificial intelligence, Computability

## Abstract

Is ethics a computable function? Can machines learn ethics like humans do? If teaching consists in no more than programming, training, indoctrinating… and if ethics is merely following a code of conduct, then yes, we can teach ethics to algorithmic machines. But if ethics is not merely about following a code of conduct or about imitating the behavior of others, then an approach based on computing outcomes, and on the reduction of ethics to the compilation and application of a set of rules, either a priori or learned, misses the point. Our intention is not to solve the technical problem of machine ethics, but to learn something about human ethics, and its rationality, by reflecting on the ethics that can and should be implemented in machines. Any machine ethics implementation will have to face a number of fundamental or conceptual problems, which in the end refer to philosophical questions, such as: what is a human being (or more generally, what is a worthy being); what is human intentional acting; and how are intentional actions and their consequences morally evaluated. We are convinced that a proper understanding of ethical issues in AI can teach us something valuable about ourselves, and what it means to lead a free and responsible ethical life, that is, being good people beyond merely “following a moral code”. In the end we believe that rationality must be seen to involve more than just computing, and that value rationality is beyond numbers. Such an understanding is a required step to recovering a renewed rationality of ethics, one that is urgently needed in our highly technified society.

## Introduction: Teaching Ethics to Machines

We, the authors, believe that an attempt at answering our title question (*Do androids dream of being good people?*) can shed some very interesting light on our understanding of what is learning, what is ethics, what is a machine, and what is a human being. Certainly, artificial intelligence (AI) raises other important ethical questions, but in this paper we reflect, first and foremost, on what *we* can learn about ethics when we consider how to teach ethical behavior to a machine.

We have identified three fundamental problems that any implementation of machine ethics will have to face. These are not simply technical problems that can be solved with better technology. Rather, they are fundamental or conceptual problems, which ultimately concern philosophical questions, such as: what is a human being (or more generally, what is a worthy being); what is human intentional acting; and how are intentional actions and their consequences morally evaluated. We will show that attempting to answer these questions requires a kind of thinking that goes beyond programming and calculation, and that the rationality of ethical acting cannot be achieved by simply imitating the behavior of others. Also, our analysis of the limitations of any machine ethics implementation with current computational approaches (whether programmed or learned) has allowed us to glean some lessons about how to teach ethics to humans, as opposed to how to teach ethics to machines.

Our intention is not to solve the technical problem of machine ethics, but to learn something about the rationality of human ethics by reflecting on the ethics that can and should be implemented in machines. Briefly, a *computational machine* is a device that follows a set of rules to solve a given problem; within the set of computational machines, an AI computer-based system is one that is capable of *receiving and evaluating information* from its environment, and of *finding non-explicitly programmed solutions* to certain problems. If ethics were merely a problem-solving *technique* –what we do not believe–, then there would be no reason to think that it cannot be learned by a machine endowed with AI. We think human reason –and ethics as a part of it– is not limited to the ability to solve problems, but it also encompasses the capacity to identify the problems worth solving.

This article is structured as follows. In “Background: A Hot Topic of Our Time” section we present machine ethics as a hot topic of our time, in order to place our subject in historical context. In “Programmed Ethics: Isaac Asimov’s Three Laws of Robotics” section we examine three fundamental difficulties of the Three Laws of Robotics which would be common to any machine ethics implementation, because they are essentially an attempt to reduce ethics to a computable code of conduct. In “Two modalities of Machine Learning” section we explain two modalities of machine learning which will be useful for better understanding how a machine could –purportedly– “learn” ethics: learning by objective and learning by imitation (readers more versed in AI will find this less useful). In “Learned Ethics: the Moral Machine” section we describe the Moral Machine experiment, as a particular application of the learning-by-imitation scheme seen in the previous section; our analysis exposes the weaknesses of the whole approach, which is basically the reduction of ethics to the imitation of majoritarian behavior. In “Conclusion: Can Machines Learn Ethics Like Humans Do?” section we present the conclusions of this essay, hoping that our reflection on machine ethics will have furnished a better understanding of what the learning of ethics entails from a human perspective.

## Background: A Hot Topic of Our Time

Machine ethics, or computational ethics, is the part of moral philosophy concerned with ensuring ethical behavior of machines that use artificial intelligence. Reflection on machine ethics started in the past century, mainly in the context of science-fiction. But not only in that context. As early as 1987, Mitchell Waldrop advocated in *AI Magazine* for the development of the theory and practice of machine ethics, and called for us to think carefully and explicitly about the values, assumptions and purposes that intelligent machines would embody, whether or not their programmers had consciously intended them (Waldrop, 1987). He even advanced the idea that the effort to endow computers with intelligence would entail some deeper comprehension of ourselves, human beings: reflect on what intelligence really is, and reexamine our conceptions of right and wrong.

The new millennium and the advent of autonomous agents brought the urgent necessity of this philosophical reflection on AI technologies. A preliminary work on the theoretical foundations for machine ethics (Anderson et al., 2004), mainly based on utilitarian ethics, was presented in a 2004 workshop organized by the Association for the Advancement of Artificial Intelligence (AAAI). Next year, an entire AAAI workshop was devoted to machine ethics, with seminal contributions that were collected and published some years later (Anderson & Anderson, 2011).

The first decade of the millennium concluded with one of the first comprehensive works on the subject, written by Wendell Wallach and Colin Allen (2009): *Moral Machines: Teaching Robots Right from Wrong*. They start this book with a framework for understanding the issues of machine ethics, structured along two independent dimensions: autonomy and sensitivity to values. Within this framework, regions of basic “operational morality” and “full moral agency” can be identified, with many gradations of “functional morality” lying in between. The authors discuss nearly all topics relevant to machine ethics, with lots of open and unanswered questions, in a text that has become a reference in the field. In spite of the catchy subtitle, however, the authors do not intend to provide a method for implementing moral algorithms in a computational machine. They do suggest, however, very briefly at the end of the book, that “the project of designing autonomous moral agents feed back into humans’ understanding of themselves as moral agents, and of the nature of ethical theory itself”.

The second decade has seen an extraordinary increase in the number of publications, both academic and popular (Coeckelbergh, 2020), together with the birth of specialized journals and public or private initiatives in favor of ethical principles in the design of AI systems, such as the Asilomar AI Principles^1^ and the Barcelona Declaration for the proper development and usage of Artificial Intelligence in Europe,^2^ both from 2017. Governments have finally made the step of taking an interest in the subject; see, for example, the initiative of the European Parliament to address the legal status of robots and AI systems (Nevejans, 2016). In general, academics and many governments have challenged the idea that AI can itself be held accountable (Bryson et. al., 2017). A variation of the Turing Test has even been proposed –the Ethical Turing Test– to determine whether an AI machine is capable of making ethical decisions (Winfield et al., 2019). Obviously, this is highly dependent on the moral principles and reasoning of the judges themselves.

When we try to reduce ethics to computations, we implicitly assume the paradigm –with deep roots in modern philosophy, and particularly in René Descartes and David Hume– that intelligence, or reason, is essentially *a universal instrument to solve problems*. But this very notion of “computational” ethics leaves its rationality in a difficult position, since the only rational part of ethics would be the reflection on the adequate means to achieve certain ends (thus, technical or instrumental reason to *solve* problems); the rationality of the ends themselves (the values, the problems *worth* solving) would not be addressed. In order to illustrate this thesis, we will examine some popular approaches to the ethical behavior of computer systems that manifest the limitations of the notion of ethics as a computation, and thus of the whole approach to machine ethics.

Fortunately, some important philosophers of the twentieth century (Martin Heidegger, Ludwig Wittgenstein, and many others following their respective paths of thought) have challenged just the idea that human reason is essentially a sort of “computational intelligence” and have shown that reason does not (solely) consist in following rules, as Hubert Dreyfus aptly explains in his famous essay *What Computers Can't Do: The Limits of Artificial Intelligence* (Dreyfus, 1972).^3^ We believe that rationality must be seen to involve more than just computing, and that *value rationality is beyond numbers*. Such an understanding is a required step to recover a renewed rationality of ethics, one that is urgently needed in our highly technified society.

Historically, there have been three main rival versions of ethical systems or traditions in Western moral philosophy: *virtue ethics* (with origins in Aristotle and other philosophers in ancient Greek), *deontological ethics* (the main exponent of which is Immanuel Kant) and *utilitarian ethics* (chiefly developed by John Stuart Mill). Even if the IEEE’s Global Initiative on Ethics of Autonomous Intelligent Systems (IEEE, 2019) has authoritatively recommended exploring all of them, together with other different culture-based ethical systems (Buddhism, Confucianism, African Ubuntu traditions, and Japanese Shinto), the truth is that current approaches to machine ethics have been inspired mainly by deontology and utilitarianism (Segun, 2021).

Allen et al. (2005) provide a framework to understanding strategies for designing artificial moral agents: top-down and bottom-up approaches to computing ethics, which are basically the same as our programmed and learned ethics exposed below. It can be easily seen, too, the close relationship of these two approaches with respectively deontological and utilitarian ethics. The idea behind *top-down approaches* is that moral principles or theories may be used as rules for the selection of ethically appropriate actions. The main problems found here are: which set of rules to select (such as the Ten Commandments, Asimov’s Laws of Robotics, etc.) and how to solve potential conflicts between rules. On the other hand, bottom-up approaches do not (supposedly) impose a specific moral theory, but seek to provide environments in which appropriate behavior is selected or rewarded, with mechanisms that could resemble child development or biological evolution. (In our opinion, however, a moral theory is always at least implicit in the way “appropriate” behavior is judged and rewarded).

In contrast, virtue ethics, which is more akin to the other non-Western traditions (Segun, 2021), has been ignored in the realm of machine ethics. This is not accidental, since virtue ethics resists being formalized into a set of rules or computations, unlike those two more modern systems (deontology and utilitarianism) which, in a sense, respectively resemble a sort of program or a cost–benefit calculation. In this sense, virtue ethics emerges as the best approach to understanding human ethics. In fact, we think the analysis of the limitations of machine ethics presented in this paper indirectly points to the exhaustion of deontology and utilitarianism that was denounced by Alasdair MacIntyre (1981), according to whom it is the dysfunction of modern moral discourse in Western societies that calls for the rehabilitation of a renewed rationality in ethics. We are witnessing in recent decades a revival of virtue ethics in general, and among technologists in particular (Génova & González, 2016), that has encouraged a shift of paradigm which escapes the limitations of the other two approaches by focusing on *being good people*, rather than just *doing the right action* (Schmidt, 2014). This undoubtedly puts ethics well beyond what is achievable by programming and computing.

## Programmed Ethics: Isaac Asimov’s Three Laws of Robotics

**Fig. 1:**
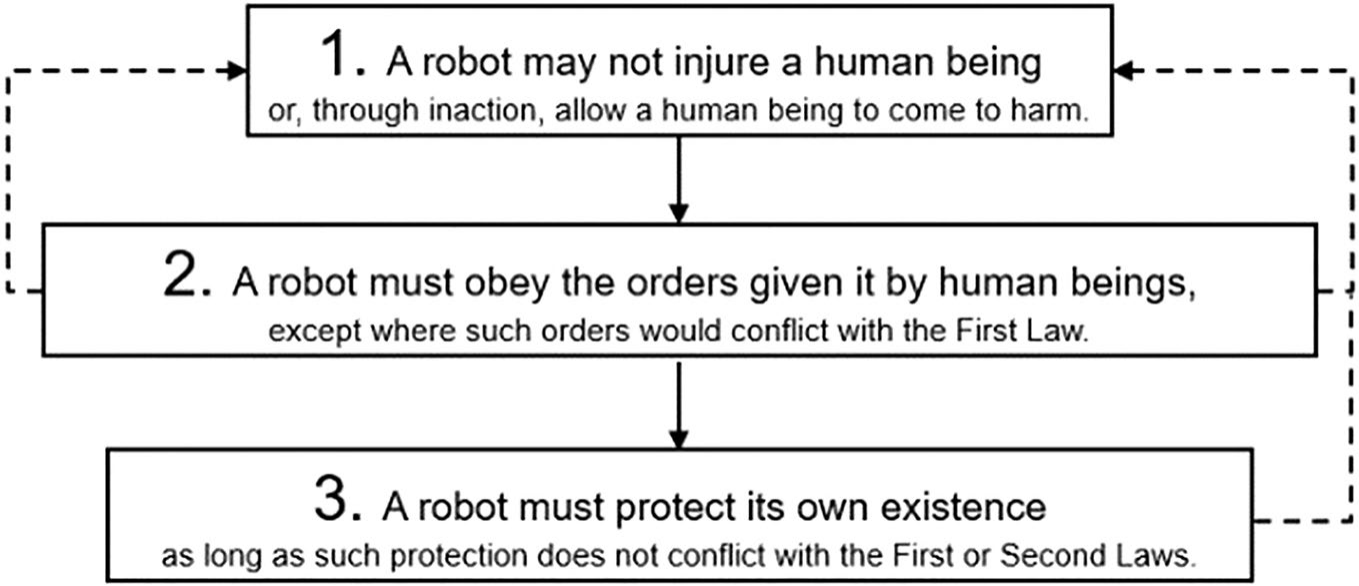
Isaac Asimov’s three laws of robotics

The ethical behavior of machines has supplied the theme for a vast amount of more or less fanciful speculation, especially in science fiction. A paradigmatic example is Isaac Asimov (1942) and his legendary Three Laws of Robotics (see Fig. 1).

The Three Laws of Robotics are a fictional attempt at explicitly programming an ethical code into a robot, i.e. a sort of *programmed ethics*. The Three Laws are certainly naïve (Lin et al., 2012), and an unsatisfactory basis for Machine Ethics (Anderson, 2011), even less for giving robots legal personality (Boden et al., 2017; Bryson, 2010; Nevejans, 2016). But it is worth examining some of the problems we would face if we were to try to implement them in a real machine. As we will see, there are a number of technical and conceptual difficulties which would be common to any machine ethics implementation, not only one that is based on the Three Laws. We will list and examine just three of these difficulties, derived from the notion of “harming a human being” or “allowing a human being to be harmed”, in the context of the First Law.

### First Difficulty: Need to Distinguish a Human from a Non-human

Since the robot has to protect and obey human beings, but not other beings, such as other humanoid robots, then it has to be able to distinguish between them properly. But this throws us into the arms of the problem that the Turing Test is seeking to address (Turing, 1950), which we still do not know how to solve, and which quite possibly can never find an algorithmical or empirical solution (Génova & Quintanilla Navarro, 2018; Northcott, 2019). Any robot implementing the Three Laws would need to incorporate as part of its basic programming a Turing Test, or some other such test, and would need to be continuously running this test with all those entities it interacts with. “That shape I see moving in the shadows of the roadside, is it a human being or is it an animal?”.

The Three Laws originally only consider members of the human species as beings worthy of respect and protection. Ought we to adapt them so that other beings, such as intelligent machines, superior animals, even extraterrestrial beings, are also considered worthy? But this would entail an additional difficulty: those other beings hypothetically included within the “ethical circle” would no longer share with humans physical, measurable properties of a biological nature (such as DNA sequence or other more easily measurable biometric characteristics). The “biological humanity test” would have to be replaced by an “ethical dignity test”.

Whether we try to base this test on external structural characteristics or else on behavioral characteristics (which is in fact the approach of the traditional Turing Test), we find that mimicking or faking the appearance and behavior of a real human (or a real “worthy being”) in order to deceive the tester is a relatively achievable task. In all wars it is common practice to use “decoys” to distract enemy attacks and render them ineffective. The attacker has to discriminate in an ever shorter time interval whether the target in sight is a soldier or a dummy, a tank or a painted wooden crate, an innocent child used as a human shield or a humanoid pretending to be a child (which would then be a legitimate military target). In other words, robotic ethical programming cannot be made viable without first solving a computational “ethical dignity test” that discriminates beings worthy of respect from other beings, in a context where we can expect to encounter faking and deception at the very least.

### Second Difficulty: Need to Foresee the Consequences of an Act

Our actions trigger a multitude of effects, some of which will occur in a more or less distant future. If only immediate effects are morally relevant, a killer robot could plan its actions in such a way that the death of the “target” will occur one week after it has made certain preparations, thereby evading responsibility for the act. If a week seems too short a time to us, shall we go for a month, or a year? Where do we draw the line?

To decide behavior in accordance with the First Law, a robot must be able to make a reasonably accurate prediction of the future. This prediction is difficult for many reasons, but especially because future events will be affected by the actions of the agent itself. Faced with the question, *is this program harmful to humans?*, an interesting recent work demonstrates, as a consequence of Rice’s theorem (Rice, 1953), that it is not possible to build a computational super-intelligence with a control strategy which forestalls evil on the part of others and which, at the same time, guarantees that it will not itself be harmful (Alfonseca et al., 2021). Like the “halting problem”, *the harming problem is undecidable*.

It is worth noting here that classical ethics, by contrast with consequentialism, does not face the same difficulty, for it places the focus of responsibility not on the *consequences* of acts, but primarily on the *intentions* which inform them (Anscombe, 1958). A reformulation of the First Law in more classical terms would be: “A robot may not *intend* to injure a human being or, through *intentional* inaction, allow a human being to come to harm”. Of course, strictly speaking, intention and consequence cannot be separated either: to act is to have the intention of producing consequences (Spaemann, 1982). But the shift of focus from the *cognitively foreseen* to the *willfully intended* is by no means negligible. What does it mean when we say that a robot has “intentions”? In other words, robotic ethical programming cannot be solved without first solving the problems of volition, intentionality and free will; in short, without solving the problem of subjectivity: of being a subject that knows and conducts him or herself—or itself.

### Third Difficulty: Need to Evaluate the Consequences as Good or Bad

This evaluation will require either an absolute classification between good and bad consequences or, at the least, a placement on a relative scale of comparison that makes some consequences preferable to others. This evaluation, implemented in computational terms, must be expressed numerically, so that a decision algorithm can be programmed to compute a result, i.e. a certain course of action. The “ethical value function” would then yield a numerical value that could be compared for each course of action considered, and thus the optimal one is chosen. In the end, it all boils down to “greater than or equal to”.^4^

Let us take the case of a “child-care robot” (such as appears in several of Asimov’s stories) that must accompany its little human in household activities. Not only must it decide whether the pleasure of playing is greater or lesser than that of eating a sweet, but it must also consider which is better from an educational point of view, from a health point of view, etc. These are different dimensions of value, and in order to combine them the typical recourse is to use rather *arbitrary coefficients* through which a sort of “weighted average” of the various goods and values at stake is achieved.

Going even further to the heart of the matter: how do we distinguish primary good and evil, that is, how do we assess the value of each of the elements involved in the formula? Is the assessment empirically verifiable, so that it could be “measured” by some kind of apparatus, and the resulting data used as input for a decision algorithm? Can dignity be measured with electrodes?

—oOo—

In short, any programmed ethical system will face, in one way or another, these three difficulties: *how to recognize* the beings worthy of respect, *how to predict* the consequences of actions, and *how to value* actions and their consequences. In other words, explicit ethical programming faces questions which our entire tradition of ethical thinking has not been able to resolve reliably, let alone translate into a “robotic” code of conduct, programmable and capable of passing quality control.

Nevertheless, AI techniques could perhaps contribute something of interest to the area of the third difficulty. Let us see how.

## Two modalities of Machine Learning

Classical programming explicitly describes the step-by-step procedure for solving a problem. The problem has been previously analyzed, it is known how to solve it, and a program, an algorithm, has been designed that can provide the solution. Thus, for example, to solve a system of two linear equations with two unknowns there is a method that can be precisely described as a sequence of steps, and by which the solution is infallibly reached, assuming it exists.

### Learning by Objective

**Fig. 2:**
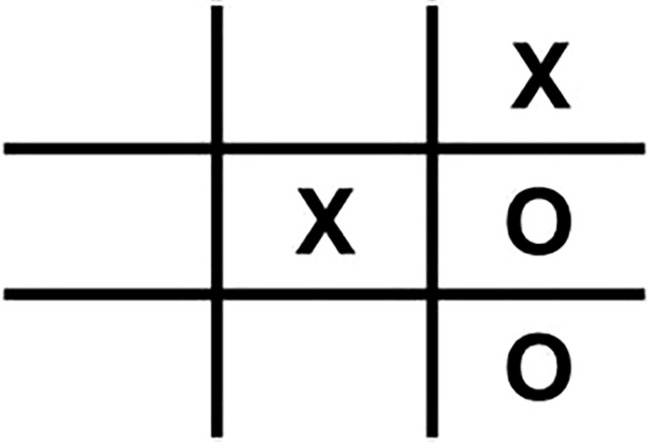
It is easy to program a machine to “learn” to play tic-tac-toe

Sometimes, however, we do not know a priori how to solve a problem, nor are we able to describe the solution procedure in a simple way. Board games are a good example. One of the simplest is tic-tac-toe (see Fig. 2). It is so simple that the strategy for a best-player a priori explicit program is within reach of the average programmer. But we can use it also as an example for a learning algorithm. One possible way to learn to play tic-tac-toe is to explore the “solution space”, i.e., all the possible combinations of moves that can occur in a game. Each of these combinations is marked according to the final result, so that retrospectively it can be known whether a given move leads to victory, defeat or draw (or is still undefined depending on subsequent moves). In this way, once the “learning” process is completed (i.e. the exploration of the solution space), we will have found *the best possible strategy* without the need to have programmed it explicitly (knowing that in tic-tac-toe there is no winning strategy: if the two players play in the best possible way, the most that can be achieved is a draw).

Chess is an infinitely more complicated game, because the multitude of possible moves leads to a combinatorial explosion that cannot be computationally analyzed. Still, something similar, though not equally effective, can be done: without coming anywhere close to exploring the entire solution space, the computer can keep a historical record of the games it has played, and mark the positions it passes through as winning, losing, or uncertain. This “knowledge” will not be definitive, because a position labeled until then as a winning one may lead in another game to defeat, in which case it reverts to “uncertain” status. Moreover, we could assess the degree of uncertainty of a position by the number of games won and lost from it, and use this ever-changing information in the decision algorithm of each game.^5^ After playing millions of games, the computer will probably not be ready to face top-level human players yet, but it will be ready to take on more modest ones.

Both cases, while differing in their ability to explore the solution space, have something in common. The “machine learning” process is capable of exploring various strategies to achieve an objective, and of selecting the best one. But all this is based on the premise that *the objective is known*, and that it is possible to determine whether it has been achieved: this is why this process can be called *learning by objective*. In both tic-tac-toe and chess there is no doubt about what is a victory and what is a defeat, and that the objective of the game is to win. *But what happens when the objective itself is not so clear?*

### Learning by Imitation

Before attempting to answer this last question, let us examine another possible form of machine learning in chess. Instead of looking at whether this or that move leads to victory or defeat, let us look at how grandmasters play. Examining the games of many players we can achieve a statistical characterization of behavior that leads to victory with a certain probability, although perhaps not infallibly. Note that in this form of *learning by imitation* we do not need to know what the objective of the game is, nor do we even need to know how to distinguish victory from defeat. All we need is information about the moves made by those who are considered good players, in order to imitate them, and those who are bad players, so as to avoid their mistakes.^6^

There are already machine learning techniques in AI that precisely bring into play this learning by imitation. Consider an automatic grading system for high school students’ literary essays. Suppose we know how to extract certain measurable features from those essays, such as spelling and grammatical correctness, readability (based on word and sentence length), argumentative correctness (let us assume this can be measured), originality of ideas (textual plagiarism), and so on. We know how to measure these features, but we do not know how to combine them into a single number—the arbitrary coefficients we mentioned above (see “Second Difficulty: Need to Foresee the Consequences of an Act”).

On the other hand, suppose we have the numerical evaluations given to these texts by a group of expert teachers. What can we do? The ingenious technique developed by AI theorists consists of randomly generating the coefficients and varying them according to certain rules until the formula gives results that conform to what the majority of experts say. *The objective*, in this case, *is to imitate* the behavior of the reference subjects, the so-called expert teachers.

For the technique to be successful it is not even necessary for teachers to be able to give a numerical value to each essay. There is no need to make the definition of “literary quality” explicit. It is enough that they are able to compare the essays two by two and say which one is better. If enough comparisons are available, the automatic learning system is able to extract a formula for the rating of each individual essay, which expresses the notion of quality that is implicit in the two-by-two comparisons made by the experts. The technique is remarkably generalizable. Experts can be asked to listen to two pieces of music and say which is more relaxing. Or to compare two faces and say which is more attractive. Or to compare two courses of action and say which is more ethical…

## Learned Ethics: the Moral Machine

One of the difficulties faced by explicit ethical programming, as previously noted (see Sect. 2.3), is recognizing right and wrong for their own sake. If the goal of ethical behavior were well defined, then perhaps we could implement a learning-by-objective process that would discover effective strategies for achieving it. However, whether or not the goal is well defined, the learning-by-imitation technique seems to open up a new possibility for the automatic learning of ethical behavior.^7^

Attempting to understand ethics as an a priori code which is capable of contemplating all possible cases is quite problematic (Lumbreras, 2017; Nallur, 2020; Torresen, 2018). That is why, in contrast to the *programmed ethics* we talked about earlier (Asimov's Three Laws), another approach has emerged in recent years, which we can call *learned ethics*. At MIT (the Massachusetts Institute of Technology) they have developed an experiment to “learn how to make machines moral”. They have named the project *The Moral Machine*: “We show you moral dilemmas, where a driverless car must choose the lesser of two evils, such as killing two passengers or five pedestrians”.^8^

### Teaching Machines to Make Decisions

The learning technique used in this project is nothing more than an application of the learning-by-imitation scheme we have seen previously (see “Second Difficulty: Need to Foresee the Consequences of an Act” section). But the same type of techniques and way of reasoning developed here could be applied to solve many other different moral dilemmas: who to select for a job, who to grant parole to, who to choose as a recipient of a transplanted organ, etc. To address the dilemmas that autonomous vehicles face, the researchers try to learn from the answers that ordinary people would give to these questions.

The idea is as follows. Since we don’t know how to explicitly program the code of ethics of an autonomous vehicle, let’s ask people: what would you do? In this situation. And in this other one. And so on. Information is extracted from the set of answers, until a set of patterns of behavior emerges. Something similar is done in other domains: as we don’t know how to program a set of explicit rules for recognizing a human face, we ask people to recognize faces; we also ask them to distinguish if this one is smiling, if this one is angry, sad or worried. Let us try then to apply these well-known techniques to the task of extracting moral knowledge from people’s responses.

**Fig. 3:**
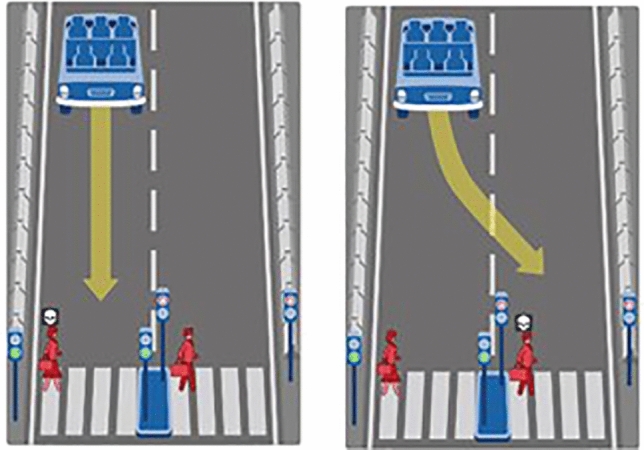
Illustration of a dilemma faced by autonomous cars: who to run over?

In each scenario, a simple dilemma is posed. We see a concrete example in Fig. 3: who should be hit by the autonomous vehicle, the woman or the man? And so on with various situations, some more complex than others, but always in the form of a simple dilemma A vs. B: men vs. women, old people vs. children, passengers vs. pedestrians, etc. The dilemma is simple, not because it is easy to solve, but because only two possible options are offered for its resolution. Like saying which of two literary essays is better.

### Problems of Machine Learning Applied to Ethical Issues

*Explainability* In machine learning the end result is a formula, an algorithm, for the recognition of face, gesture or handwriting. But it is not possible to explain *why it works*. There is no other justification for the formula beyond its effectiveness—in other words, the fact that it has a very high success rate, expressed in its high concordance with the answers of “the reference experts”, i.e. the surveyed population itself. Yet this is hardly a surprise, since learning algorithms are designed and tested specifically to achieve a high success rate. And of course, when the decision carries a heavy ethical charge, the fact that one cannot provide reasons for it is a serious, a very serious problem (Wallach & Allen, 2009; Bryson, 2019).

*Bias* Ethical algorithms –so it is said– have to avoid bias, in whatever form it may take. Biases against women, against African Americans, against those who dress unconventionally, etc. But then, what if the population we are surveying in the experiment is itself biased? The bias must be avoided, no matter what the majority says. This highlights a very interesting point: we know (believe?) that bias, or being biased, is a bad thing. In other words, *good and evil is not what the majority says*, it is beyond that. This is not an original consideration: it is at the very origins of ethical reflection in Greek philosophy. What is interesting is that AI brings this consideration back into focus.

*Sample selection* A third, closely related problem, is the selection of people to whom we ask: who would you run over? That is, who are we asking, ordinary people or sadists? And why shouldn’t sadists be included in the survey? Aren’t we biasing the sample? And how do we know who are ordinary people, and who are sadists? If avoiding sadists is a concern, and if we think we can recognize them, this means that we are applying an ethical criterion already in the selection of the sample: it is a priori, it is previous to the experiment. Even if we do not know it perfectly, we already know, in a way, what is good and what is bad before doing the experiment.

### Discussion: Ethics, Majorities and Critical Thinking

In short, our claim is that ethics does not consist in the imitation of typical or majority behavior. We humans do not teach ethics like that. If a national or regional government were to include in its educational programs an ethical approach such as the following: “boys and girls must imitate the majority”… would we not rebel with tremendous indignation? The very seed of ethics is critical thinking, a refusal to conform to the dominant mindset.

Many distinctions between the terms ‘ethics’ and ‘morality’ have been proposed, although none has achieved universal consensus (Gert & Gert, 2020). One of the most common distinctions assigns morality a “descriptive” sense (social mores or codes of conduct), whereas ethics is assigned a “normative” sense (what is *actually* right or wrong, beyond whatever is socially accepted). In this sense, the Moral Machine is certainly effective in its reflection of social mores, but it will never be a true *Ethical Machine*.

The approach taken by the Moral Machine has been strongly criticized (Bremner et al., 2019; Nascimento et al., 2019; Puri, 2020). The problem of autonomous cars should not be whom to kill, but how not to kill (Holstein et al., 2021). Etienne discusses the dangers of the Moral Machine experiment; he objects both to using it for normative ends and to the whole voting system approach it is built upon in addressing ethical issues (Etienne, 2020). Jaques goes so far as to call the Moral Machine “a monster” (Jaques, 2019), because it actually invites people to express preferences –i.e. biases– for external indicators of *social value*. This is objectionable, she argues, not only because humans are very bad at judging people based on appearances, but especially because the Moral Machine suggests and even advocates for the moral relevance of those features—which is nothing less than a direct attack on personal dignity.

Ethics is not an imitation game (Génova et al., 2022). Ethics is not about following a code of conduct like Asimov’s Three Laws. But neither is ethics about imitating the behavior of others. Learning by imitation is something very human, but if there is any *ethical* difference between doing A and doing B, that difference is not in the number of people who behave one way or another, but in the *reasonableness* and *good will* of their behavior.

## Conclusion: Can Machines Learn Ethics Like Humans Do?

We are convinced that a proper understanding of ethical issues in AI can teach us something valuable about ourselves, and what it means to lead a free and responsible ethical life, beyond merely “following a moral code”. It is perfectly valid to argue that algorithms should reflect ethical values; but to claim, as some do, that ethical values can be translated into numerical values through which good and evil would become “computable” (i.e., calculable by some kind of mathematical formula), reflects a tremendously impoverished understanding of ethics and of human life; a life whose aim is not merely to “do the right thing”, but to grow towards a goal –a fullness– that is not predetermined, and which consequently cannot be measured or verified; an open fullness which we have to discover and conquer through the living of life itself and the recognition of the other, who can never be totally encompassed (Génova & González, 2017). Such an essential ingredient of a virtuous life –of being a good person– is not programmable, because a program, in a way, is closed to novelty. Table 1 summarizes –as five pairs of Don’ts and Do’s– the lessons learned about how to teach ethics to humans when we consider how to teach ethical behavior to a machine.

**Table 1:** Don'ts and Do’s in teaching ethics to humans

Do not try to program the behavior of people, do not indoctrinate: rules may help, but ethics has to look beyond the rules	Do teach to *recognize* good, value, dignity: good behavior comes naturally after
Do not focus exclusively on consequences	Do emphasize intention and explainability: consider *wanted* and *unwanted* consequences
Do not focus on measuring and putting numbers to things and values	Do exercise thinking *beyond numbers*, beyond programming and calculation
Do not teach simple, closed dilemmas: ethics is not a geometry problem-solving technique; dilemmas can serve as an initiation, although they cannot be the only form of teaching ethics	Do teach to discover new ways to solve problems, beyond predefined solutions; *focus on being a good person*, rather than just doing the right action
Do not teach only by imitation	Do encourage *creativity*: invent new forms of doing good and being good

We have seen that AI provides ingenious “learning” techniques to solve problems whose method of resolution is not known a priori, or is not easy to describe in a simple way. However, the final result of the machine learning process is the same as when using explicit programming: *mechanical behavior according to a code of rules*, whether this code is programmed (top-down) or “learned” (bottom-up).

Machine learning is an effective method of training an algorithmic machine, but it is far from the genuine educational aspiration to form ethical thinking in human beings, which should include the formation of conscience, of one’s own critical sense, beyond mere imitation of the behavior of others and beyond mere “problem solving”. There are several levels in human pedagogy: conditioning, instruction, training, formation and education; but the first three are not adequate contexts to teach ethics, since that would be considered indoctrination.

An approach based on computing outcomes, and on the reduction of ethics to the compilation and application of a set of rules, either a priori or learned, represents a serious misconception of ethics. Above all, the attempt to formalize ethics in a set of rules misses the point that *a person is not only an instance of a case*, but a unique and unrepeatable being. A person is a child, a student, a patient, a client, a neighbor (*your* child, *your* student, *your* patient, *your* client, *your* neighbor). A person’s value cannot be measured with a number, not even with an array of numbers (age, sex, health status, social contribution…).

We need to recover value rationality beyond numbers, beyond logical and instrumental rationality. We need to learn how to reason with values in a way that does not convert them into numbers. The first commandment of ethics should be “thou shalt not treat a person as *a vector of numbers*”. And if this demand requires a renewed rationality of ethics, then so be it!

So then, can machines learn ethics like humans do?

By now, the answer should be obvious. If *teaching* consists in no more than programming, training, indoctrinating… and if *ethics* is merely following a code of conduct, then yes, you can teach ethics to algorithmic machines.

The interesting thing about this final question is that it reveals something about *what teaching is* and *what ethics is*. For teaching is not simply training (although training in various skills is part of the educational process), nor is ethics about following a code of conduct (although codes of conduct play a very important role in ethics). Perhaps robots can learn to do *the right thing* (and it is certainly important that they do), but ethical life goes beyond that. Because, at its core, ethical life is the intimate aspiration to be *good people*, and this requires recognizing and responding to the novelty of the other. Do androids dream of being good people?^9^

## Footnotes

1 https://futureoflife.org/open-letter/ai-principles/

2 https://www.iiia.csic.es/barcelonadeclaration/

3 The essay was re-titled in 1992, two decades after the first edition, as “What Computers *Still* Can't Do. A Critique of Artificial Reason”.

4 This includes those situations when certain robotic action would harm a group of humans, but inaction would harm another group. The *lesser of two evils principle* is at the heart of the analysis of the Trolley Problem (Foot, 1967; Thomson, 1976) that in turn serves as a model for the Moral Machine (see  "Two modalities of Machine Learning").

5 This technique is known as “reinforcement learning”, but what we have described here is a rough simplification of it. The number of possible configurations of pieces on a chessboard is so high that such simple statistics of the value of a certain configuration will hardly be meaningful. A real learning algorithm will consider abstract states, each of which will represent a much broader set of concrete configurations.

6 The attentive reader will have noticed that learning by imitation is still a type of learning by objective. The only difference is that the objective is no longer to win the game, but to imitate as perfectly as possible the game of the good players.

7 This section is a brief synthesis of the explanations given in (Génova et al., 2022). We think this synthesis is necessary to maintain the integrity of our argument and to develop it further.

8 *The Moral Machine* (https://www.moralmachine.net/). In this project, the results of an impressive number of respondents have been analyzed: 40 million responses from people in 233 countries in ten different languages (Awad et al., 2018). In a previous paper (Bonnefon et al., 2016), the authors have shown that survey subjects tend to prefer utilitarian algorithms for autonomous vehicles (maximization of the number of lives saved, even at the cost of the passengers); but, contradictorily, they disapprove enforcing utilitarian regulations (they would themselves prefer to ride vehicles that protect their passengers at all costs).

9 *Do Androids Dream of Electric Sheep?* is the title of the original 1968 novel by Philip K. Dick that inspired the 1982 film *Blade Runner* directed by Ridley Scott.
